# Spectral kissing and its dynamical consequences in the squeeze-driven Kerr oscillator

**DOI:** 10.1038/s41534-023-00745-1

**Published:** 2023-07-28

**Authors:** Jorge Chávez-Carlos, Talía L. M. Lezama, Rodrigo G. Cortiñas, Jayameenakshi Venkatraman, Michel H. Devoret, Victor S. Batista, Francisco Pérez-Bernal, Lea F. Santos

**Affiliations:** 1 Department of Physics, University of Connecticut, Storrs, CT 06269 USA; 2 Department of Physics, Yeshiva University, New York, NY 10016 USA; 3 Department of Applied Physics and Physics, Yale University, New Haven, CT 06520 USA; 4 Department of Chemistry, Yale University, P.O. Box 208107, New Haven, CT 06520-8107 USA; 5 Departamento de Ciencias Integradas y Centro de Estudios Avanzados en Física, Matemáticas y Computación, Universidad de Huelva, Huelva, 21071 Spain; 6 Instituto Carlos I de Física Teórica y Computacional, Universidad de Granada, Fuentenueva s/n, 18071 Granada, Spain

**Keywords:** Quantum simulation, Qubits

## Abstract

Transmon qubits are the predominant element in circuit-based quantum information processing, such as existing quantum computers, due to their controllability and ease of engineering implementation. But more than qubits, transmons are multilevel nonlinear oscillators that can be used to investigate fundamental physics questions. Here, they are explored as simulators of excited state quantum phase transitions (ESQPTs), which are generalizations of quantum phase transitions to excited states. We show that the spectral kissing (coalescence of pairs of energy levels) experimentally observed in the effective Hamiltonian of a driven SNAIL-transmon is an ESQPT precursor. We explore the dynamical consequences of the ESQPT, which include the exponential growth of out-of-time-ordered correlators, followed by periodic revivals, and the slow evolution of the survival probability due to localization. These signatures of ESQPT are within reach for current superconducting circuits platforms and are of interest to experiments with cold atoms and ion traps.

## Introduction

Recent developments in superconducting circuits have opened the pathway to explore long standing predictions of quantum physics. They have been used to study dynamical bifurcation^1,2^, to squeeze quantum fluctuations^3^, to prepare exotic quantum states, and to process and stabilize quantum information^4,5^. Here, we propose to use this platform as a quantum simulator of excited state quantum phase transitions (ESQPTs), a phenomenon that occurs in various nuclear, atomic, molecular, and condensed matter systems. The superconducting circuit considered is a driven system, whose static effective Hamiltonian describes a double-well system and thus exhibits an ESQPT. This perspective adds another layer of interest to the long history of studies on driven nonlinear oscillators^6–14^, where the emergence of a double well, reached by driving the oscillator at twice its original frequency^6^, has been explored in studies of quantum activation^6,7^, quantum tunneling^8,9^, and the preparation of selected superpositions of quasienergy states^10^ with applications to quantum information science, such as the generation of Schrödinger cat states.

A quantum phase transition (QPT) corresponds to an abrupt change in the ground state of a physical system when a control parameter reaches a critical point. It occurs in the thermodynamic limit, but scaling analyses of finite systems can signal its presence. ESQPT is a generalization of this phenomenon to excited states^15–18^, which can take place independently of the presence of QPTs^19,20^ and can be triggered by anharmonicities^21–23^. In an ESQPT, the separation of the states in two phases^24^ occurs at a point that depends on both the value of the energy and of the control parameter. There is a vast literature on the subject, which is reviewed in ref. ^18^. ESQPTs are associated with enhanced decoherence^25,26^, localized eigenstates^27–29^, very slow^27–29^ or accelerated^30–32^ quantum quench dynamics, specific dynamical features at long times^33–35^, isomerization reactions^36^, and the creation of Schrödinger cat states^20^.

The main signature of an ESQPT is a singularity in the density of states (DOS) that moves to higher excitation energies as the control parameter increases, and may be accompanied by the closing of energy gaps between excited states. The energy where the divergence of the DOS takes place is the ESQPT critical energy. These and related features have been theoretically identified in various quantum systems with few degrees of freedom^15–49^, and a proposal to detect the ESQPT with spinor Bose–Einstein condensates also exists^50^.

Even though spectroscopic signatures of the ESQPT have been experimentally observed^51–55^ and its presence suggested from the bifurcation phenomenon detected in refs. ^56–58^, presently none of these systems provides the means to analyze the spectrum as a function of the control parameter and to simultaneously observe the dynamical consequences of an ESQPT in a controllable way. Superconducting circuits close this gap by offering a platform that has an experimental realizable classical limit and provides both frequency- and time-resolved high quantum non-demolition measurements fidelity^59^.

As we explain here, the exponential approach of pairs of adjacent levels (spectral kissing) recently observed in the spectrum of the superconducting Kerr resonator as a function of the amplitude of a squeezing drive^59^, and previously discussed in^10^, marks the presence of an ESQPT. The dynamical counterpart of this transition presents a seeming paradoxical behavior, which can, in principle, be observed in a system such as the one in ref. ^59^. For Glauber coherent states close to the ESQPT, the initial decay of the survival probability (overlap of the initial and the evolved state) is slower than for coherent states away from the ESQPT, while the fidelity out-of-time-ordered correlator (FOTOC) grows exponentially fast for the first and slower for the latter. The justification for these apparently opposite behaviors lies in the classical limit of the system. At the origin of the phase space, (*q* = 0, *p* = 0), there is a stationary but unstable point that is associated with the ESQPT. At this point, the evolution is dominated by the squeezing part of the Hamiltonian.

The experimental capability of reconstruction of the full phase-space distribution^59^ motivates our analysis of the dynamics in phase space, which reveals features that were missed by previous works on ESQPTs and that are of interest to studies of nonequilibrium quantum dynamics. Depending on the initial state, the exponentially fast spread in phase space can be followed by the onset of complicated interference patterns or yet by periodic revivals that persist for long times. Our analysis also elucidates why states with exactly the same energy may exhibit different dynamics.

## Results

### Quantum system

The system that we investigate was implemented in a superconducting circuit^59^ based on driven SNAIL^60^ transmons. The static effective Hamiltonian of this system is given by (Supplementary Note 1)

1 $$\frac{{\hat{H}}_{qu}}{\hslash \,K}=\hat{n}(\hat{n}-1)-\xi \left({\hat{a}}^{{\dagger} 2}+{\hat{a}}^{2}\right),$$

where $\hat{n}={\hat{a}}^{{\dagger} }\hat{a}$, *K* is the Kerr nonlinearity, *ξ* = *ϵ*_2_/*K* is the control parameter, and *ϵ*_2_ is the squeezing amplitude. The system conserves parity, $[{\hat{H}}_{qu},{(-1)}^{{\hat{a}}^{{\dagger} }\hat{a}}]=0$.

**Fig. 1:**
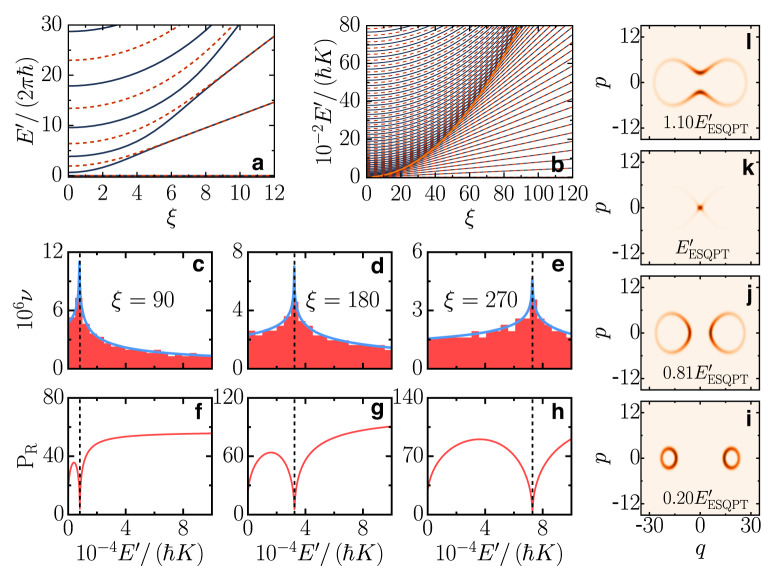
Spectral kissing and localization. **a** Energy levels as a function of the control parameter reproducing the experimental data^59^ with *K*/(2*π*) = 0.32 MHz and **b** ${E}^{{\prime}}/(\hslash K)$ for larger values of *ξ*. Solid lines are for the even parity sector and dashed lines for odd parity. The bright orange line in (**b**) marks the energy of the ESQPT, as given in Eq. (3). **c–e** Normalized density of states and **f–h** participation ratio for the eigenstates in the Fock basis for the values of *ξ* indicated in (**c–e**); even parity sector. Numerical (shade) and analytical (solid line) data are shown in (**c–e**). The vertical dashed line in (**c–h**) is the ESQPT energy from Eq. (3). **i–l** Husimi functions for different eigenstates and *ξ* = 180.

We study the spectrum of ${\hat{H}}_{qu}$ as a function of the control parameter *ξ* in Fig. 1a–e. The plots display the excitation energies, ${E}^{{\prime} }=(E-{E}_{0})$, where *E* are the eigenvalues of ${\hat{H}}_{qu}$ and *E*_0_ its ground state energy. The numerical data in Fig. 1a reproduce the experimental data in Fig. 3A of ref. ^59^. One sees that as the control parameter increases, the coalescence of a pair of adjacent eigenvalues, each level belonging to a different parity sector, happens at a higher energy. This spectral kissing becomes better visible in Fig. 1b, where larger values of *ξ* are used. For a given value of the control parameter, the spectral kissing happens at the critical energy of the ESQPT, ${E}_{{{{\rm{ESQPT}}}}}^{{\prime} }$, which is marked with a solid line in Fig. 1b and is obtained analytically [see Eq. (3) below].

In addition to the exponential approach of the energies in each pair, the eigenvalues cluster at ${E}_{{{{\rm{ESQPT}}}}}^{{\prime} }$ (Supplementary Note 2). This produces the peak of the DOS displayed for different values of the control parameter in Fig. 1c–e. The peak diverges for *ξ* → *∞*, which is a main signature of the ESQPT^17^.

The presence of the ESQPT gets reflected in the structure of the eigenstates, $\left\vert \psi \right\rangle ={\sum }_{n}{C}_{n}\left\vert n\right\rangle$, written in the Fock basis, ${\hat{a}}^{{\dagger} }\hat{a}\left\vert n\right\rangle =n\left\vert n\right\rangle$. The eigenstates at the vicinity of the ESQPT are highly localized in the Fock state $\left\vert 0\right\rangle$^27–29^. This can be quantified with the participation ratio, ${{{{\rm{P}}}}}_{{{{\rm{R}}}}}=1/\mathop{\sum }\nolimits_{n = 0}^{{{{\mathcal{N}}}}-1}| {C}_{n}{| }^{4}$, where ${{{\mathcal{N}}}}$ is the size of the truncated Hilbert space. P_R_ is large for an extended state and small for a localized state. In Fig. 1f–h, we show the participation ratio as a function of ${E}^{{\prime} }$. An abrupt dip in the value of P_R_ happens for ${E}^{{\prime} } \sim {E}_{{{{\rm{ESQPT}}}}}^{{\prime} }$ and the analysis of the components of the eigenstate at this energy confirms its localization at $\left\vert 0\right\rangle$. Equivalently to *P*_R_, the plot of the occupation number $\langle \psi | {\hat{a}}^{{\dagger} }\hat{a}| \psi \rangle$ as a function of energy exhibits a dip at ${E}^{{\prime} } \sim {E}_{{{{\rm{ESQPT}}}}}^{{\prime} }$ (Supplementary Note 2).

The localization at the ESQPT critical point is also detected with the Husimi function^61^ obtained by writing the eigenstates in the basis of Glauber coherent states [see Eq. (13)]. The Husimi function gives the distribution of the quantum state in the phase space of canonical variables (*q*, *p*). As seen in Fig. 1k, the eigenstate closest to the ESQPT energy is highly concentrated in the origin of the phase space. This contrasts with the eigenstates below the ESQPT [Fig. 1i, j], which present two separated ellipses, and the eigenstates above it [Fig. 1l]. The localization in the phase space mirrors the localization in the Fock basis, since the coherent state with (*q* = 0, *p* = 0) coincides with the Fock state $\left\vert 0\right\rangle$.

### Classical limit

The Hamiltonian of the Kerr oscillator in Eq. (1) develops two wells when *ξ* > 0. The depth of the wells and their energy levels grow as *ξ* increases, bringing the system closer to the classical limit. Experimentally, the value of *ξ* can be increased by reducing the impedance of the circuit, increasing the microwave power of the squeezing drive, or approaching the Kerr-free point (Supplementary Note 1).

The grounds for the onset of the ESQPT are found in the classical limit. The classical Hamiltonian is derived in Methods and is given by

2 $$\frac{{H}_{cl}}{K}=\frac{1}{4}{({q}^{2}+{p}^{2})}^{2}-\xi ({q}^{2}-{p}^{2}).$$

**Fig. 2:**
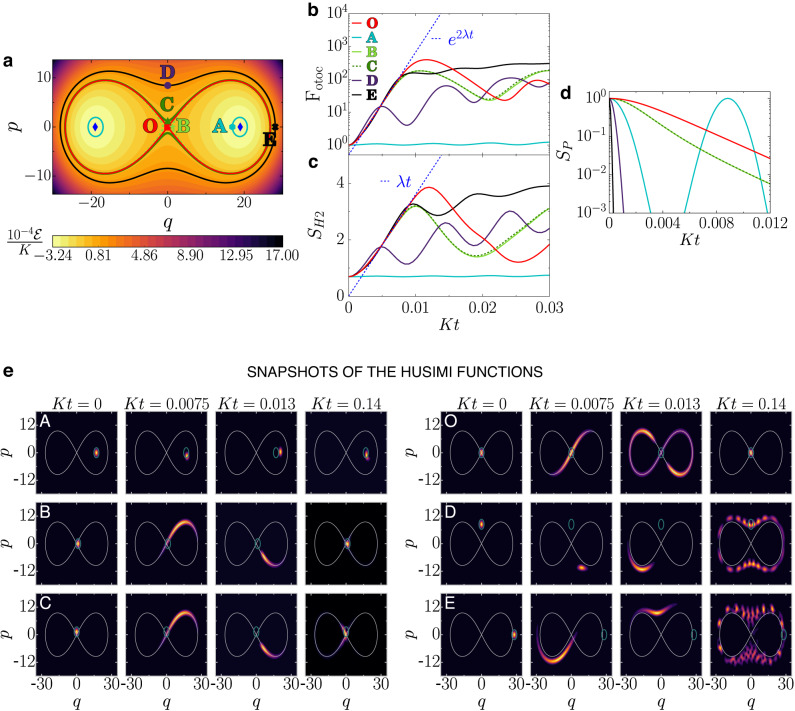
Phase space and quantum dynamics. **a** Energy curves in the phase space obtained with Eq. (2). The hyperbolic point is denoted as O, the center points are represented with blue diamonds, and the solid line intersecting at O is the separatrix. Points O, A–E mark the centers of the initial coherent states chosen for the quantum dynamics. **b** Evolution of the FOTOC, **c** Husimi entropy, and **d** survival probability as a function of time. The exponential [linear] curve with rate [slope] given by the Lyapunov exponent in Eq. (4) are indicated in (**b**)[**c**]. **e** Snapshots of the Husimi functions; each row refers to one of the six initial coherent states investigated, and each column to a different time, as indicated.

It presents three stationary points when *ξ* > 0. They are the two center points $\{q,p\}=\{\pm \sqrt{2\xi },0\}$ with the minimal energy of the system ${{{{\mathcal{E}}}}}_{\min }={H}_{cl}(q,p)=-K{\xi }^{2}$, and the hyperbolic point {*q*, *p*} = {0, 0} with energy ${{{{\mathcal{E}}}}}_{{{{\rm{hyp}}}}}=0$. In the plot of the energy contours in Fig. 2a, the hyperbolic point is indicated as O, the red line that intersects at this point is the separatrix, and the two blue diamonds are the center points.

The properties of the quantum system find a parallel in the classical limit. The energy difference ${{{{\mathcal{E}}}}}_{{{{\rm{hyp}}}}}-{{{{\mathcal{E}}}}}_{\min }$ marks the separatrix in Fig. 2a and determines the energy of the ESQPT,

3 $${E}_{{{{\rm{ESQPT}}}}}^{{\prime} }\approx K{\xi }^{2},$$

which is indicated with a bright orange line in Fig. 1b. The equality in Eq. (3) holds in the classical limit. Below this energy, the pairs of stable periodic orbits with equal energy are analogous to the degenerate states of the quantum system, and above that line the degeneracy is lost. The stationary point at the origin of the phase space, (*q*, *p*) = (0, 0), justifies the localization at the Fock state $\left\vert 0\right\rangle$ of the eigenstate with energy at the ESQPT.

The existence of a non-degenerate hyperbolic point implies the logarithmic discontinuity of the level density, as shown in refs. ^18,62^, and explains the peak at ${E}_{{{{\rm{ESQPT}}}}}^{{\prime} }$ in Fig. 1c–e. Using the smooth component of the Gutzwiller trace formula^63^, we obtain a semiclassical approximation for the DOS (Supplementary Note 3). This curve outlines the numerical data in Fig. 1c–e.

Another consequence of the hyperbolic point is the onset of a positive Lyapunov exponent (Supplementary Note 4),

4 $$\lambda =2K\xi .$$

The system described by Eq. (2) is regular, so the Lyapunov exponent for any initial condition is zero, except for the unstable point O^32,64,65^.

### Quantum dynamics: instability

The instability associated with the hyperbolic point is manifested in the quantum domain with the exponential growth of out-of-time-ordered correlators (OTOCs)^32,64–66^. These quantities, defined as ${O}_{{{{\rm{toc}}}}}=\langle {[\hat{W}(t),\hat{V}(0)]}^{2}\rangle$, measure the spread (scrambling) of quantum information by assessing how the operators $\hat{W}$ and $\hat{V}$ fail to commute due to the evolution of $\hat{W}$^67^. A particular example of OTOCs is the FOTOC, which corresponds to having the operator $\hat{V}=\left\vert \Psi (0)\right\rangle \left\langle \Psi (0)\right\vert$, for the initial state $\left\vert \Psi (0)\right\rangle$, and $\hat{W}={{{{\rm{e}}}}}^{i\delta \phi \hat{G}}$, where *δϕ* is a small perturbation and $\hat{G}$ is a Hermitian operator. In the perturbative limit, *δϕ* ≪ 0, the FOTOC is the variance ${\sigma }_{G}^{2}(t)=\langle {\hat{G}}^{2}(t)\rangle -{\langle \hat{G}(t)\rangle }^{2}$^68^.

We analyze the evolution of the FOTOC given by the variance of *p* and *q*,

5 $${F}_{{{{\rm{otoc}}}}}(t)={\sigma }_{p}^{2}(t)+{\sigma }_{q}^{2}(t),$$

because the initial coherent states that we consider spread in both canonical coordinates^32^. These states are centered at the points O, A–E, marked in Fig. 2a, and are denoted as $\left\vert {\Psi }_{j}(0)\right\rangle$ with *j* = *O*, *A*, …, *E*. State $\left\vert {\Psi }_{A}(0)\right\rangle$ has the lowest energy, followed by $\left\vert {\Psi }_{B}(0)\right\rangle$ (negative energy close to zero), $\left\vert {\Psi }_{O}(0)\right\rangle$ (zero energy), and $\left\vert {\Psi }_{C}(0)\right\rangle$ (positive energy close to zero). States $\left\vert {\Psi }_{D}(0)\right\rangle$ and $\left\vert {\Psi }_{E}(0)\right\rangle$ have equal and high positive energy (see Methods).

We compare the growth of *F*_otoc_(*t*) in Fig. 2b with the Husimi entropy,

6 $${S}_{{{{\rm{H}}}}2}(t)=-\ln {M}_{2}(t),$$

in Fig. 2c, where *M*_2_(*t*) is the integral of the square of the Husimi function (Supplementary Note 5.1). Both quantities, *F*_otoc_(*t*) and *S*_H2_(*t*), measure how an evolving state spreads in the phase space. Snapshots of the evolution of the Husimi functions for $\left\vert {\Psi }_{A,B,C}(0)\right\rangle$ (left) and for $\left\vert {\Psi }_{O,D,E}(0)\right\rangle$ (right) are presented in Fig. 2e (more snapshots are in Supplementary Note 5.1 and videos are available in^69^). The results are as follows.

(O): After the parabolic increase in *t*, that happens for very short times $Kt \,< \,K\tau ={(\sqrt{8}\xi )}^{-1}$ (Supplementary Note 6), ${F}_{{{{\rm{otoc}}}}}^{(O)}(t)$ [${S}_{{{{\rm{H}}}}2}^{(O)}(t)$] for the initial coherent state at the hyperbolic point, $\left\vert {\Psi }_{O}(0)\right\rangle$, grows exponentially [linearly] fast with a rate proportional to the classical Lyapunov exponent given in Eq. (4), that is, ${F}_{{{{\rm{otoc}}}}}^{(O)}(t)\propto {{{{\rm{e}}}}}^{2\lambda t}$ [${S}_{{{{\rm{H}}}}2}^{(O)}(t)\propto \lambda t$]. The snapshot of the Husimi function for a time as small as *Kt* = 0.013 indicates that $\left\vert {\Psi }_{O}(t)\right\rangle$ is already very spread out in phase space, covering an area larger than that for the other five states, even those with larger energies. Indeed, around *Kt* = 0.013, ${F}_{{{{\rm{otoc}}}}}^{(O)}(t)$ [${S}_{{{{\rm{H}}}}2}^{(O)}(t)$] reaches the highest value among the states considered, as seen in Fig. 2b[c]. The maximum value happens at the Ehrenfest time, ${{{\mathcal{T}}}} \sim \ln (\xi )/\lambda$ (Supplementary Note 7).

The fast scrambling of quantum information for $\left\vert {\Psi }_{O}(t)\right\rangle$, which happens for $\tau \,<\, t \,< \,{{{\mathcal{T}}}}$, is later followed by partial reconstructions of the initial distribution (see the Husimi function at *Kt* = 0.14). In the absence of dissipation, this yo-yo process of spreading and contraction persists for a long time (Supplementary Note 5.2). This behavior is the quantum counterpart of the classical dynamics at the vicinity of the hyperbolic (saddle) point O, which is both a repellor and an attractor (Supplementary Note 4), resulting in trajectories that move both towards and away from O. We also note that despite reaching the highest value at $t \sim {{{\mathcal{T}}}}$, the infinite-time average of ${F}_{{{{\rm{otoc}}}}}^{(O)}(t)$ is actually smaller than the saturation value for ${F}_{{{{\rm{otoc}}}}}^{(D,E)}(t)$ (Supplementary Note 7). This result shows that the degree of spreading quantified by OTOCs depends not only on the initial state and system, but also on the timescale.

(A): The initial coherent state $\left\vert {\Psi }_{A}(0)\right\rangle$ is very close to a center point, so the evolution is very slow, ${F}_{{{{\rm{otoc}}}}}^{(A)}(t)$ [${S}_{{{{\rm{H}}}}2}^{(A)}(t)$] never reaches large value, and the Husimi function remains close to the point A.

(B) and (C): State $\left\vert {\Psi }_{B}(0)\right\rangle$ [$\left\vert {\Psi }_{C}(0)\right\rangle$] is slightly below [above] the ESQPT. Instead of the confinement around the center point imposed to the classical orbit B, quantum effects allow $\left\vert {\Psi }_{B}(t)\right\rangle$ to escape and evolve similarly to $\left\vert {\Psi }_{C}(t)\right\rangle$. The spread of the Husimi distributions for both states is comparable, reaching regions of the phase space with + *q* and − *q* (see snapshots in Fig. 2e and in Supplementary Note 5.2). In addition, since B and C are in the vicinity of the unstable point O, quantum fluctuations trigger the exponential [linear] growth of ${F}_{{{{\rm{otoc}}}}}^{(B,C)}(t)$ [${S}_{{{{\rm{H}}}}2}^{(B,C)}(t)$] observed in Fig. 2b, c. This behavior is at odds with the classical limit, where the positive Lyapunov exponent emerges only at the hyperbolic point and not close to it. As *ξ* increases and one approaches the classical limit, the duration of the exponential behavior for ${F}_{{{{\rm{otoc}}}}}^{(B,C)}(t)$ decreases.

(D) and (E): States $\left\vert {\Psi }_{D}(0)\right\rangle$ and $\left\vert {\Psi }_{E}(0)\right\rangle$ have the same high energy, but evolve differently. In terms of scrambling, $\left\vert {\Psi }_{E}(0)\right\rangle$ combines the best of both worlds, because in addition to high energy, which leads to the largest saturation value for ${F}_{{{{\rm{otoc}}}}}^{(D,E)}(t)$ (Supplementary Note 7), it partially overlaps with the separatrix (see the snapshot of the Husimi function at *t* = 0 in Fig. 2e), so ${F}_{{{{\rm{otoc}}}}}^{(E)}(t)$ [${S}_{{{{\rm{H}}}}2}^{(E)}(t)$] in Fig. 2b, c presents an exponential [linear] growth analogous to that seen for $\left\vert {\Psi }_{B,C}(0)\right\rangle$, which is absent for $\left\vert {\Psi }_{D}(0)\right\rangle$. The spread of the Husimi distribution for $\left\vert {\Psi }_{E}(0)\right\rangle$ happens simultaneously inside and outside the separatrix (Supplementary Note 5.2), leading to complicated quantum interference effects, as those observed in the snapshot of the Husimi function at *Kt* = 0.14.

### Quantum dynamics: localization

While the fastest and longest scrambling happens for the initial coherent state $\left\vert {\Psi }_{O}(0)\right\rangle$, this state also presents the slowest decay of the survival probability,

7 $${S}_{p}(t)={\left\vert \langle \Psi (0)| \Psi (t)\rangle \right\vert }^{2}.$$

The survival probability for all other initial coherent states, with energy above or below the ESQPT, decays faster than ${S}_{p}^{(O)}(t)$, as seen in Fig. 2d.

The apparent paradox of the fast spread of $\left\vert {\Psi }_{O}(t)\right\rangle$, as measured by ${F}_{{{{\rm{otoc}}}}}^{(O)}(t)$ and ${S}_{{{{\rm{H}}}}2}^{(O)}(t)$, and the slow decay of ${S}_{p}^{(O)}(t)$ is naturally resolved in view of the classical limit and from the analysis of the Husimi functions. The instability associated with the hyperbolic point O is the source of the exponentially fast spread of the variance of the phase-space distribution, but O is also a stationary point (the gradient of the Hamiltonian at this point is zero), so $\left\vert {\Psi }_{O}(0)\right\rangle$ is strongly localized in the eigenstate at the ESQPT [see Fig. 1k]. In other words, the width of the energy distribution for $\left\vert {\Psi }_{O}(0)\right\rangle$, given by $\sqrt{2}K\xi$, is the smallest one among the six states (Supplementary Note 6). Close to the origin of the phase space, the evolution is dominated by the squeezing, ${\hat{H}}_{qu}\approx {\epsilon }_{2}({\hat{q}}^{2}-{\hat{p}}^{2})$. This leads to the rapid stretching of $\left\vert {\Psi }_{O}(t)\right\rangle$, while part of the population remains for some time in the vicinity of the origin. These two aspects of the dynamics become evident in the snapshot of the Husimi function for $\left\vert {\Psi }_{O}(t)\right\rangle$ at *Kt* = 0.0075. The small green ellipse in those panels indicates the size of the initial coherent state. One sees that the Husimi distribution for $\left\vert {\Psi }_{O}(t)\right\rangle$ at *Kt* = 0.0075 is stretched out, but part of it remains inside the green ellipse.

## Discussion

This work bridges communities working on superconducting circuits, ESQPTs, and nonequilibrium quantum dynamics. The squeeze-driven Kerr oscillator is an addition to the list of nuclear, molecular, and condensed matter systems that exhibit ESQPTs. Its advantage is to be experimentally realizable in an available superconducting circuit platform, where both frequency and time domain measurements can be done simultaneously, the control parameter can be tuned to approach the classical limit, arbitrary initial states can be prepared, and the dynamics can be studied in phase space. We expect superconducting circuits to become versatile quantum simulators for ESQPTs and related phenomena, such as isomerization, where the separation between neighboring energy levels decreases close to the isomerization barrier height^70,71^.

The dynamical consequences of ESQPTs that we presented should also appeal to experimental platforms, where long-range couplings can be tuned to approach models with collective interactions, such as those with cold atoms^72^ and trapped ions^73^. Of interest to those experiments is the demonstration of the exponential growth of OTOCs, which we showed to emerge for different initial states placed close to the separatrix that marks the ESQPT. Other highlights include the later revivals of a coherent state initially centered at the phase-space origin, the combined effects of fast scrambling and subsequent interferences for a high-energy state close to the separatrix, and the different dynamics for states with the same energy but initially located in different regions of the phase space.

We conclude with a brief discussion about the static effective Hamiltonian, ${\hat{H}}_{qu}$, investigated here and used to describe the driven SNAIL transmon in ref. ^59^. As the drive amplitude and nonlinearities of the experimental system increase, ${\hat{H}}_{qu}$ ceases to be valid, the ESQPT melts away, and chaos eventually sets in. The emergence of chaos, which could be captured experimentally and may affect the development of quantum devices, cannot be described by any static effective Hamiltonian^9,14,59^ obtained for systems with only one degree of freedom. The analysis of chaos, which will be the subject of our forthcoming papers, has to rely entirely on the original time-dependent Hamiltonian.

## Methods

In the Supplementary Note 1, we describe how the original driven Hamiltonian leads to the static effective Hamiltonian,

8 $$\frac{{\hat{H}}_{qu}}{\hslash }=-K{\hat{a}}^{{\dagger} 2}{\hat{a}}^{2}+{\epsilon }_{2}({\hat{a}}^{{\dagger} 2}+{\hat{a}}^{2}),$$

and how the parameters can be experimentally controlled. In the main text, we changed the sign of the Hamiltonian in Eq. (1) for convenience, so that we could say that *E*_0_ in ${E}^{{\prime} }=E-{E}_{0}$ is the ground state energy of ${\hat{H}}_{qu}$, instead of its highest energy. Regardless of the sign convention, dissipation will bring the experimental system to the attractors (stable nodes) in the bottom of the wells, which define unambiguously the ground state of the system.

### Classical limit

For large values of the control parameter, *ξ* = *ϵ*_2_/*K* ≫ 1, the double wells created by the quantum Hamiltonian in Eq. (8) become very deep and the number of levels inside the wells become macroscopic, so ${\hat{H}}_{qu}$ exhibits properties comparable to the classical Hamiltonian. However, to derive the classical Hamiltonian for any depth of the wells, that is, to approach a continuous spectrum for a fixed and not necessarily large value of the control parameter, we introduce the parameter *N*_eff_, whose reciprocal is related with the size of the zero point fluctuations. We write

9 $$\hat{a}=\sqrt{\frac{{N}_{{{{\rm{eff}}}}}}{2}}\,\left(\hat{q}+i\hat{p}\right),$$

and

$$[\hat{q},\hat{p}]=\frac{i}{{N}_{{{{\rm{eff}}}}}},$$

so the classical limit can be reached by taking *N*_eff_ → ∞, since $\hat{q}\to q$ and $\hat{p}\to p$. This way, the quantum Hamiltonian,

10 $$\begin{array}{ll}\displaystyle\frac{{H}_{qu}}{\hslash }=-\frac{K{N}_{{{{\rm{eff}}}}}^{2}}{4}{\left(\hat{q}-i\hat{p}\right)}^{2}{\left(\hat{q}+i\hat{p}\right)}^{2}\\\qquad\quad\displaystyle +\;\xi \frac{K{N}_{{{{\rm{eff}}}}}}{2}[{\left(\hat{q}-i\hat{p}\right)}^{2}+{\left(\hat{q}+i\hat{p}\right)}^{2}],\end{array}$$

leads to the classical Hamiltonian (with *ħ* = 1),

11 $${H}_{cl}=-\frac{{K}_{cl}}{4}{({q}^{2}+{p}^{2})}^{2}+{K}_{cl}{\xi }_{cl}({q}^{2}-{p}^{2}),$$

where

$$K={K}_{cl}/{N}_{{{{\rm{eff}}}}}^{2}\quad {{{\rm{and}}}}\quad \xi ={\xi }_{cl}{N}_{{{{\rm{eff}}}}}.$$

In the main text, we fixed

$${N}_{{{{\rm{eff}}}}}=1,$$

and used large values of *ξ*.

The experimental system admits an approximate classical description if it is initialized in a coherent state and for as long as the Hamiltonian phase space surface produces only a linear force (a quadratic Hamiltonian) over the spread of the evolving state.

### Husimi function

For an eigenstate written in the basis of the Glauber coherent states,

12 $$\left\vert \alpha \right\rangle ={{{{\rm{e}}}}}^{-\frac{1}{2}| \alpha {| }^{2}}\mathop{\sum }\limits_{n=0}^{{{{\mathcal{N}}}}}\frac{{\alpha }^{n}}{\sqrt{n!}}\left\vert n\right\rangle ,$$

where $\hat{a}\left\vert \alpha \right\rangle =\alpha \left\vert \alpha \right\rangle$, ${{{\mathcal{N}}}}$ is the truncation of the Hilbert space,

$$\alpha =\sqrt{\frac{1}{2}}(q+ip)$$

and *N*_eff_ = 1, the Husimi function is given by

13 $${Q}^{\psi }(q,p)=\frac{1}{2\pi }{\left\vert \mathop{\sum }\limits_{n = 0}^{{{{\mathcal{N}}}}}{C}_{n}{{{{\rm{e}}}}}^{-\frac{({q}^{2}+{p}^{2})}{4}}\frac{{(q-ip)}^{n}}{\sqrt{{2}^{n}n!}}\right\vert }^{2}.$$

### Initial coherent states

The six initial coherent states that we consider are obtained by using in Eq. (12) the values of *p* and *q* specified below. These are the points marked in Fig. 2a. Their classical energies ${{{\mathcal{E}}}}$ are given for *ξ*_*cl*_ = 180.

14 $$\begin{array}{rcl}\,{{\mbox{Point O}}}\,:&&q=0,p=0,\\ &&{{{\mathcal{E}}}}/{K}_{cl}=0.\\ \,{{\mbox{Point A}}}\,:&&q=16.9143,p=0,\\ &&{{{\mathcal{E}}}}/{K}_{cl}=-3.1034\times 1{0}^{4}.\\ \,{{\mbox{Point B}}}\,:&&q=1.2533,p=0,\\ &&{{{\mathcal{E}}}}/{K}_{cl}=-0.0282\times 1{0}^{4}.\\ \,{{\mbox{Point C}}}\,:&&q=1.2506,p=0,\\ &&{{{\mathcal{E}}}}/{K}_{cl}=0.0282\times 1{0}^{4}.\\ \,{{\mbox{Point D}}}\,:&&q=0,p=8.4443,\\ &&{{{\mathcal{E}}}}/{K}_{cl}=1.4106\times 1{0}^{4}.\\ \,{{\mbox{Point E}}}\,:&&q=28.1302,p=0,\\ &&{{{\mathcal{E}}}}/{K}_{cl}=1.4106\times 1{0}^{4}.\end{array}$$

### Supplementary information

Supplementary Information

## Data Availability

All data for Fig. 1 and Fig. 2 can be downloaded from https://www.dropbox.com/scl/fi/0tggwm9wyjiknrwmx1o8x/DATA_npjQuantInf.zip?rlkey=4stxzad21bmk7fijh79yiwc6a&dl=0or from https://gitlab.com/currix1/kerr_resonator_animations.

## Code availability

All the computational codes that were used to generate the data presented in this paper are available from the corresponding authors upon request.
