# Supplementary material for: Spectral kissing and its dynamical consequences in the squeeze-driven Kerr oscillator
Source: npj Quantum Inf. 2023 Jul 28;9(1):76. doi: 10.1038/s41534-023-00745-1 (PMC11041765; doi:10.1038/s41534-023-00745-1)
Supplement: Supplementary file 1 — Supplementary Information [file 41534_2023_745_MOESM1_ESM.pdf]

Supplementary Information: Spectral kissing and its dynamical consequences in the squeeze-driven Kerr oscillator

Jorge Chávez-Carlos,[1] Talía L.M. Lezama,[2] Rodrigo G. Cortiñas,[3] Jayameenakshi Venkatraman,[3]
Michel H. Devoret,[3] Victor S. Batista,[4] Francisco Pérez-Bernal,[5,6] and Lea F. Santos[1]

[1]*Department of Physics, University of Connecticut, Storrs, Connecticut 06269, USA*
[2]*Department of Physics, Yeshiva University, New York, New York 10016, USA*
[3]*Department of Applied Physics and Physics, Yale University, New Haven, Connecticut 06520, USA*
[4]*Department of Chemistry, Yale University, P.O. Box 208107, New Haven, Connecticut 06520-8107, USA*
[5]*Departamento de Ciencias Integradas y Centro de Estudios Avanzados en Física, Matemáticas y Computación, Universidad de Huelva, Huelva 21071, Spain*
[6]*Instituto Carlos I de Física Teórica y Computacional, Universidad de Granada, Fuentenueva s/n, 18071 Granada, Spain*

This Supplementary Information is organized as follows. Supplementary Note 1 contains details about the quantum and classical Hamiltonians of the squeeze-driven Kerr oscillator and a discussion about the experimental parameters. Supplementary Note 2 compares two plots that present the excitation energies E' as a function of the control parameter ξ. In one plot both parities are included and in the other one, only one parity sector is considered. In addition, this Supplementary Note includes a figure for the occupation number, which also detects the ESQPT. Supplementary Notes 3 and 4 give the density of states (DOS) and the Lyapunov exponent, respectively. Supplementary Note 5 provides an equation for the integral of the square of the Husimi function and additional snapshots for the evolution of the Husimi functions for the six initial coherent states studied in the main text. In Supplementary Note 6, we discuss how to derive the time interval for the initial quadratic behavior in t of the survival probability, FOTOC, and $M_2(t)$. Supplementary Note 7 shows the duration of the exponential growth of the FOTOC for the coherent state O and the saturation values of the FOTOC for the six initial coherent states that we study.

Supplementary Note 1. QUANTUM AND CLASSICAL HAMILTONIANS

In the same way that an LC circuit is the electrical analog of a mechanical harmonic oscillator, the Josephson junction is the electrical analog of a mechanical pendulum. The Hamiltonian of a single Josephson junction is [1, 2]

$$\hat{H} = \frac{1}{2C}\hat{Q}^2 - E_J \cos\left(\frac{2\pi}{\Phi_0}\hat{\Phi}\right),$$

where C is the circuit's capacitance, E_J is the Josephson energy, $\hat{\Phi}$ is the phase circuit variable, and $\hat{Q}$ its charge, with $[\hat{\Phi}, \hat{Q}] = i\hbar$ [2]. This is the canonical commutation relation that describes quantum circuits and is analogous to the position-momentum relation in a mechanical system. The charge enters the Hamiltonian as a quadratic kinetic energy and the circuit's phase enters via the Josephson cosine potential and is analogous to the projection of a constant gravitational field over the vertical as in a pendulum potential [3].

One defines the bosonic operators of the circuit as a convenient calculation tool. The annihilation operator for a superconducting circuit takes the form

$$\hat{a} = \sqrt{\frac{1}{2\hbar Z}}\left(\hat{\Phi} + iZ\hat{Q}\right) \tag{1}$$

where Z is the impedance of the circuit and $[\hat{a}, \hat{a}^\dagger] = 1$. Alternatively, one can write

$$\hat{\Phi} = \Phi_{\rm zpf}\left(\hat{a}^\dagger + \hat{a}\right), \quad \hat{Q} = iQ_{\rm zpf}\left(\hat{a}^\dagger - \hat{a}\right), \tag{2}$$

where $\Phi_{\rm zpf} = \sqrt{\hbar/2\omega C} = \sqrt{\hbar Z/2}$ is the zero point spread of the phase variable, ω is the small oscillation frequency of the oscillator, and $\Phi_{\rm zpf}Q_{\rm zpf} = \hbar/2$. Insisting on the parallel with the mechanical oscillator, $\Phi_{\rm zpf}$ is the electrical analog to the ground state position uncertainty and $Q_{\rm zpf}$ corresponds to the ground state momentum uncertainty. The capacitance C then plays the role of the particle's mass.

In the case of the SNAIL transmon used in Ref. [4], the Hamiltonian of the driven circuit, which is built by an

arrangement of a few Josephson junctions, reads

$$\frac{\hat{H}(t)}{\hbar} = \omega\hat{a}^\dagger\hat{a} + \sum_{m=3}^{\infty}\frac{g_m}{m}\left(\hat{a}^\dagger + \hat{a}\right)^m \\ - i\Omega_d\left(\hat{a} - \hat{a}^\dagger\right)\cos(\omega_d t). \tag{3}$$

This is Eq. (1) in Ref. [4], where the g_n's are the circuit nonlinearities and the drive is defined by its amplitude Ω_d and its frequency ω_d, which is fixed at two times the small oscillation frequency of the oscillator to create resonant squeezing. Since nonlinearity is sourced by an arrangement of Josephson junctions in the SNAIL, the g_n coefficients are of order $\Phi_{\rm zpf}^{n-2}$ [5]. Additionally, the magnetic flux tuning of a SNAIL permits the tunability of the oscillator's nonlinearities [6]. In particular, one can tune the values of $g_3(\Phi_B)$ and $g_4(\Phi_B)$ rather accurately. For the sake of this discussion, we will approximate the impedance of the circuit as independent from the magnetic flux.

The static effective Hamiltonian describing the system in these conditions is given by

$$\frac{\hat{H}}{\hbar} = -K\hat{a}^{\dagger 2}\hat{a}^2 + \epsilon_2(\hat{a}^{\dagger 2} + \hat{a}^2), \tag{4}$$

where, from the microscopic theory introduced in [6], we can write the Kerr constant as $K = -\frac{3g_4}{2} + 2\frac{10g_3^2}{3\omega_d}$ and $\epsilon_2 = g_3\frac{4\Omega_d}{3\omega_d}$ [4]. This Hamiltonian is the quantum optical analog of a double-well potential [7] and the number of levels inside the wells is given by $N = \xi/\pi$ [4], where $\xi = \epsilon_2/K$ is the control parameter.

The Hamiltonian in Eq. (4) can be factorized to read

$$\hat{H} = -K(\hat{a}^{\dagger 2} - \epsilon_2/K)(\hat{a}^2 - \epsilon_2/K).$$

This means that the coherent states $|\pm\alpha\rangle$, with $(\pm\alpha)^2 = \sqrt{\epsilon_2/K}$, are both degenerate with eigenenergy zero. Since $-\hat{H}$ is positive semidefinite, then $|+\alpha\rangle$ and $|-\alpha\rangle$ are degenerate ground states of the system and can be thought of as the ground states of the double well. Note that the bonding and antibonding superpositions $\propto (|+\alpha\rangle \pm |-\alpha\rangle)$ are exactly degenerate for all well-depths. This implies that there is no tunnel splitting between the well ground-states. This is a peculiarity of our Hamiltonian that has important consequences for the dynamics [8, 9]. Beyond the ground state, it is only in the classical limit $\xi \to \infty$, that the degeneracy is total for the excited states. For finite values of ξ, the excited state splitting is reduced exponentially with ξ.

We note that flux tuning a SNAIL circuit allows for a Kerr-free point [10], where the Kerr constant is null. We express this condition by writing $K = \Phi_{\rm zpf}^2\kappa(\Phi_B)$, with $\kappa(\Phi_B)$ a function crossing zero. In addition, the third-order nonlinearity responsible for the generation of squeezing remains essentially constant in the vicinity of the Kerr-free point, so we can write the scaling of the control parameter ξ as a function of the experimentally controllable variables,

$$\xi = \frac{\epsilon_2}{K} \propto \frac{\Omega_d}{\Phi_{\rm zpf}\kappa(\Phi_B)}. \tag{5}$$

With this expression, it is clear that the value of ξ can be increased in three different ways. One can (i) reduce the impedance of the circuit, thus reducing $\Phi_{\rm zpf}$, (ii) increase the microwave power of the squeezing drive Ω_d, or (iii) approach the Kerr-free point by in situ magnetic flux tuning Φ_B.

Supplementary Note 1.1. Classical limit

As we wrote in Methods, the experimental system admits an approximate classical description if it is initialized in a coherent state and for as long as the Hamiltonian phase space surface produces only a linear force (a quadratic Hamiltonian) over the spread of the evolving state [11]. This means that the dynamics will be generated by the Poisson bracket, since the Moyal corrections can be neglected, and no phase space interference effects will develop. This can be achieved by reducing the fluctuations of the coherent state (increasing its "mass", $\Phi_{\rm zpf} \to 0$), or by making a comparatively large double-well system ($\Omega_d/\kappa(\Phi_B) \to \infty$). Note that reducing $\Phi_{\rm zpf}$ comes at the price of increasing the spread in the momentum coordinate. In a Hamiltonian with quadratic kinetic energy, like Eq. (3), this comes at a minimal cost, since no nonlinearity is experienced along the momentum (charge) axis, and the Moyal corrections remain small. Note, however, that in the presence of a Kerr nonlinearity, the Hamiltonian has a nonlinear dependence on the momentum coordinate and the classical correspondence needs to be treated carefully. This justifies taking the classical limit as a system of increasing size, $\xi \gg 1$, as in the main text, which can be achieved independently of the value of zero point fluctuations.

In the absence of dissipation, this classical Hamiltonian approximation breaks at sufficiently long times for most initial conditions. In turn, small amounts of dissipation enforce the classical dynamics [4, 12, 13]. As in [12, 14], the system discussed here is not chaotic, but since for a state initialized near the ESQPT, the evolution can be approximated by a quadratic Hamiltonian (squeezing, $\epsilon_2 \gg K$), the exponential instability is a property of both the quantum and the classical models. The evolution can be approximated as classical until the phase space distribution folds on the quartic energy wall and develops phase interferences, such as those seen in the last snapshop of the last row of the Fig. 2e in the main text [15]. This quantum-classical divergence will be regularized in a timescale set by dissipation. The possibility to experimentally explore the quantum-classical correspondence in the squeeze-driven Kerr oscillator will be communicated elsewhere.

Supplementary Note 2. CLUSTERING OF EIGENVALUES AND STATIC OBSERVABLE

Supplementary Figure 1a is identical to Fig. 1b in the main text. Supplementary Figure 1b is the same as Supplementary Figure 1a, but displayed for a single parity sector with the purpose of making it evident that the clustering of the eigenvalues at E'_{ESQPT} happens also in a single sector. The size $\mathcal{N}$ of the truncated Hilbert space here and everywhere in this work is chosen to guarantee the convergence of the energy levels analyzed.

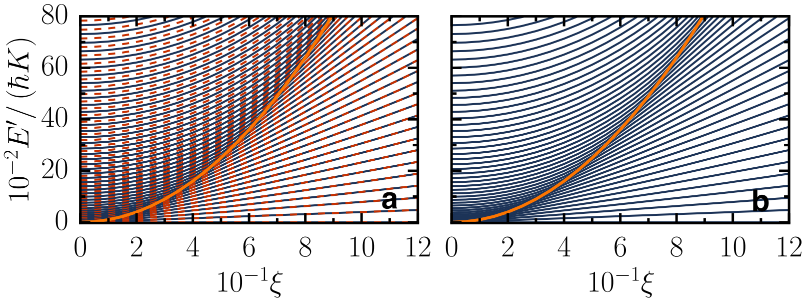

Supplementary Figure 1. Spectral kissing and eigenvalues clustering. **a** Energy levels as a function of the control parameter ξ for both parity sectors and **b** for the even parity only. In **a**: solid lines are for the energy levels in the even parity sector and dashed lines for the odd parity. The bright solid line in both panels marks the energy of the ESQPT, as given in Eq. (3) of the main text.

Supplementary Figure 2 shows the eigenstate expectation value of the number operator, $\langle \hat{n} \rangle = \langle \psi | \hat{a}^\dagger \hat{a} | \psi \rangle$, as a function of the excitation energies. A dip is clearly seen at the ESQPT energy, $E' \sim E'_{\mathrm{ESQPT}}$, which is caused by the fact that the eigenstate at this energy is localized in the Fock state $|0\rangle$. The figure is analogous to that of the participation ratio in Figs. 1f-h of the main text.

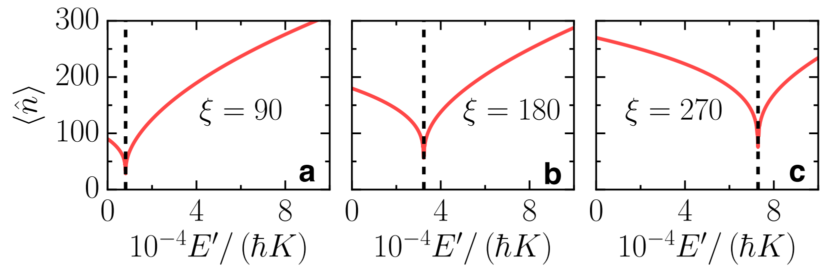

Supplementary Figure 2. Occupation number detects the ESQPT. Eigenstate expectation value of the occupation number as a function of the excitation energies for different values of the control parameter ξ, as indicated in the panels. An abrupt dip happens at $E' \sim E'_{\mathrm{ESQPT}}$.

Supplementary Note 3. DENSITY OF STATES

We can use the lowest-order term of the Gutzwiller trace formula [16] to obtain a semiclassical approximation for the DOS,

$$\nu(\mathcal{E}) \;=\; \frac{1}{2\pi}\int dpdq\delta(H_{cl}-\mathcal{E}), \tag{6}$$

where H_{cl} is given by

$$H_{cl} = -\frac{K_{cl}}{4}(q^2+p^2)^2 + K_{cl}\xi_{cl}(q^2-p^2). \tag{7}$$

This is Eq. (2) of the main text and, with the proper sign, it corresponds to Eq. (11) in Methods. To evaluate the previous integral, we use the general property of the Dirac delta,

$$\int_{\mathbb{R}^n} f(\mathbf{x})\delta(g(\mathbf{x}))d\mathbf{x} = \int_{g^{-1}(0)} \frac{f(\mathbf{x})}{|\nabla g|}d\sigma(\mathbf{x}), \tag{8}$$

where the integral on the right is over $g^{-1}(0)$ and the $(n-1)$-dimensional surface defined by $g(\mathbf{x}) = 0$. Employing the property of the Dirac delta in the Gutzwiller formula, we have

$$\nu(\mathcal{E}) = \frac{1}{2\pi}\int_{q\in\Omega_{\mathcal{E}}} \frac{dq}{2\sqrt{(2\sqrt{K_{cl}\,u(\mathcal{E})}-(\lambda+K_{cl}q^2))u(\mathcal{E})}}, \tag{9}$$

where $u(\mathcal{E}) = \mathcal{E}-\mathcal{E}_{\min}+\lambda q^2$, $\lambda = 2K_{cl}\xi_{cl}$, and $\Omega_{\mathcal{E}}$ is the set of values of q for which there is at least one solution of the equation $H_{cl}(q,p) = \mathcal{E}$.

Supplementary Note 4. LYAPUNOV EXPONENT

The linear analysis around the center and the hyperbolic points gives us information about the qualitative behavior close to those points. In particular, for the Hamiltonian in Eq. (7), the linearized Hamilton equations around a critical (stationary) point $\{q_c, p_c\}$ satisfy

$$\begin{pmatrix}\dot q\\ \dot p\end{pmatrix} = \begin{pmatrix} -2K_{cl}q_c\,p_c & -2K_{cl}\xi_{cl}-K_{cl}(q_c^2+3p_c^2)\\ -2K_{cl}\xi_{cl}+K_{cl}(3q_c^2+p_c^2) & 2K_{cl}q_c\,p_c\end{pmatrix}\begin{pmatrix}q-q_c\\ p-q_c\end{pmatrix}.$$

In the equation above, *cl* stands for "classical" and *c* for "critical". The stability or instability around $\{q_c, p_c\}$ is given by the eigenvalues a_i of the matrix constructed by the linear system. If the eigenvalues of the matrix are real, then the Lyapunov exponent is equal to $\max(a_i)$.

For the specific case of the hyperbolic point $\{q_c, p_c\} = \{0, 0\}$, the linear system is given by

$$\begin{pmatrix}\dot q\\ \dot p\end{pmatrix} = \begin{pmatrix} 0 & -2K_{cl}\xi_{cl}\\ -2K_{cl}\xi_{cl} & 0\end{pmatrix}\begin{pmatrix}q\\ p\end{pmatrix} \tag{10}$$

and the Lyapunov exponent is

$$\lambda = 2K_{cl}\xi_{cl}. \tag{11}$$

At the vicinity of the hyperbolic point, the dynamics is dominated by the squeezing part of the Hamiltonian, $H_{cl} \approx K_{cl}\xi_{cl}(\hat{q}^2-\hat{p}^2)$, and the solution of Eq. (10) gives

$$\begin{pmatrix}q\\ p\end{pmatrix} = c_1\begin{pmatrix}1\\ 1\end{pmatrix}\mathrm{e}^{\lambda t} + c_2\begin{pmatrix}-1\\ 1\end{pmatrix}\mathrm{e}^{-\lambda t}, \tag{12}$$

where c_1 and c_2 are constants. Two directions of evolution control the dynamics in the phase space, the direction $\begin{pmatrix}1\\ 1\end{pmatrix}$ is affected by the positive exponential, as a repellor, and $\begin{pmatrix}-1\\ 1\end{pmatrix}$ is affected by the negative exponential, as an attractor. The hyperbolic (saddle) point at the origin of phase space is both a repellor and an attractor. A trajectory in the vicinity of this point moves towards and away from it. The quantum counterpart of this behavior is observed with the evolution of the Husimi function for the initial coherent state centered at O={0,0}, which spreads rapidly at short times, but eventually folds back towards the initial distribution. This behavior is shown in Fig. 2e of the main text and is made yet more evident with the additional snapshots presented in the Supplementary Figure 3.

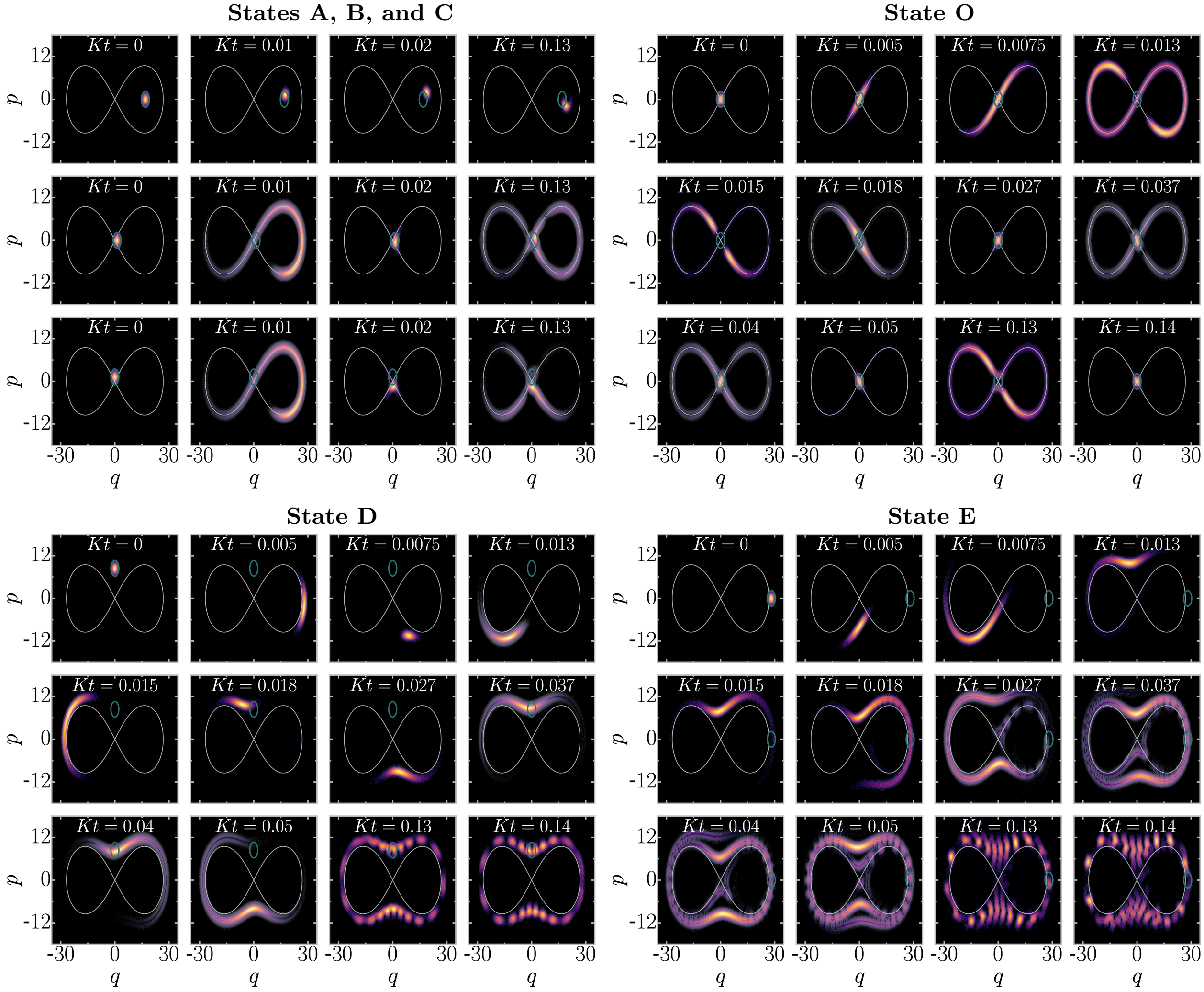

Supplementary Figure 3. Spread in phase space captured by the evolution of Husimi functions. Snapshots of the Husimi functions for the 6 initial coherent states investigated, as indicated in the titles; $\xi = \xi_{cl} = 180$. On the top left, the snapshots in the first row of panels are for state A, in the second row they are for B, and the third row for C.

Supplementary Note 5. QUANTUM DYNAMICS

The 6 initial coherent states that we consider are those listed in Eq. (14) of the Methods in the main text.

Supplementary Note 5.1. Integral of the square of the Husimi function

One can quantify how an initial coherent state spreads in the phase space by computing the integral of the square of the Husimi function,

$$M_2^{\Psi(t)} = \frac{1}{2\pi} \int dq\, dp\, [Q^{\Psi(t)}(q,p)]^2, \tag{13}$$

where $N_{\text{eff}} = 1$ and

$$Q^{\Psi(t)}(q,p) = \frac{1}{2\pi} \left| \sum_{n=0}^{\mathcal{N}} C_n(t) \mathrm{e}^{-\frac{(q^2+p^2)}{4}} \frac{(q-ip)^n}{\sqrt{2^n n!}} \right|^2 . \tag{14}$$

By writing the evolved state in the Fock basis, $|\Psi(t)\rangle = \sum_n C_n(t)|n\rangle$, one can solve the integrals exactly and obtain

$$M_2^{\Psi(t)} = \frac{1}{\pi} \sum_{n_1,n_2,m_1,m_2} \frac{C_{n_1}(t)C^*_{n_2}(t)C_{m_1}(t)C^*_{m_2}(t)}{\sqrt{n_1!n_2!m_1!m_2!}} \int d^2\alpha\, \mathrm{e}^{-2|\alpha|^2} \alpha^{*\,n_1+m_1} \alpha^{n_2+m_2}$$
$$= \sum_{n_1,n_2,m_1,m_2} \frac{C_{n_1}(t)C^*_{n_2}(t)C_{m_1}(t)C^*_{m_2}(t)}{\sqrt{n_1!n_2!m_1!m_2!}} \frac{(n_1+m_1)!}{2^{n_1+m_1+1}} \delta_{n_1+m_1,n_2+m_2} \,. \tag{15}$$

Supplementary Note 5.2. Snapshots of the evolution of the Husimi functions

Here, we present various snapshots of the evolution of the Husimi functions for the 6 initial coherent states investigated. The main features are summarized below.

The three rows of panels on the top left of Supplementary Figure 3 present snapshots of the Husimi functions for four instants of time for the initial coherent states $|\Psi_A(0)\rangle$ (first row), $|\Psi_B(0)\rangle$ (second row), and $|\Psi_C(0)\rangle$ (third row). State A has very low energy and thus exhibits a very limited spreading around its initial region in the phase space. At $Kt = 0.02, 0.13$, the distribution gets mostly out of the green ellipse that determines the initial state, so the value of $S_p^{(A)}(t)$ should become very small.

In contrast to $|\Psi_A(t)\rangle$, the Husimi distributions for $|\Psi_B(t)\rangle$ and $|\Psi_C(t)\rangle$ get squeezed, but do not fully leave the green ellipse. These two states present evolutions similar to the coherent state $|\Psi_O(t)\rangle$, since the two also start close to the hyperbolic point at the origin of the phase space. As mentioned in the main text, the fact that $|\Psi_B(t)\rangle$ evolves towards the region with negative values of q is a quantum effect. The classical point B has a positive value of q and is inside the separatrix, so classically, its orbit never reaches values of $q < 0$.

The various snapshots of the Husimi functions for $|\Psi_O(t)\rangle$ (top right), $|\Psi_D(t)\rangle$ (bottom left), and $|\Psi_E(t)\rangle$ (bottom right) complement those displayed in the main text. The panels for $|\Psi_O(t)\rangle$ make evident the fast spread of this state, and also the subsequent alternating spread and contraction of its Husimi function.

State E also spreads fast, because it is placed on the separatrix, although far from the origin. Just as for B and C, its exponential behavior is a quantum effect. Part of the quantum evolution of the coherent state E happens inside the separatrix and part of it is outside, creating two spreading fronts, as visible from the snapshots at $Kt = 0.027, 0.037, 0.04$, and 0.05. These different paths generate a complicated pattern of interferences, as shown for $Kt = 0.13$ and 0.14.

Quantum interferences also appear for the initial coherent state $|\Psi_D(0)\rangle$. This state has a high energy that is equal to that of state E, but since $|\Psi_D(0)\rangle$ starts far from the separatrix, it does not spread as fast as $|\Psi_E(0)\rangle$; compare their Husimi functions, for example, at $Kt = 0.037, 0.04, 0.05$.

Supplementary Note 6. QUADRATIC BEHAVIOR IN TIME

At very short times, the survival probability, FOTOC, and $M_2(t)$ present a quadratic behavior in time. The time interval for this behavior is derived by doing a Taylor expansion of the propagator $U(t) = \mathrm{e}^{-i\hat{H}t}$, as discussed next.

Supplementary Note 6.1. Survival probability

At short times, the survival probability, can be written as [17]

$$\begin{aligned} S_p(t) &= \left| \langle \Psi(0)|\mathrm{e}^{-i\hat{H}t}|\Psi(0)\rangle \right|^2 \\ &\approx \left| \left\langle \Psi(0) \middle| 1 - i\hat{H}t - \frac{\hat{H}^2 t^2}{2} \middle| \Psi(0) \right\rangle \right|^2 \\ &= 1 - t^2 \left[\langle \Psi(0)|\hat{H}^2|\Psi(0)\rangle - \langle \Psi(0)|\hat{H}|\Psi(0)\rangle^2 \right] \\ &= 1 - \Gamma^2 t^2, \end{aligned}$$

where Γ^2 is the variance of the energy distribution of the initial state written in the energy eigenbasis, that is

$$\Gamma^2 = \sum_k |C_k^{(0)}|^2 (E_k^2 - E_0)^2,$$

where $\hat{H}|E_k\rangle = E_k|E_k\rangle$, $E_0 = \langle\Psi(0)|\hat{H}|\Psi(0)\rangle$, and

$$C_k^{(0)} = \langle E_k|\Psi(0)\rangle.$$

Using the Fock basis $|n\rangle$ to write Γ_O^2 for the initial coherent state O, we have that

$$\begin{aligned}\Gamma_O^2 &= \sum_n \langle 0|\hat{H}|n\rangle\langle n|\hat{H}|0\rangle - \langle 0|\hat{H}|0\rangle^2 \\ &= \sum_{n\neq 0} \left|\langle n|\hat{H}|0\rangle\right|^2,\end{aligned}$$

therefore,

$$S_p^O(t) \approx 1 - 2\xi^2 K^2 t^2. \tag{16}$$

This implies that the survival probability for the state O decays quadratically for

$$Kt < \frac{1}{\sqrt{2}\xi}. \tag{17}$$

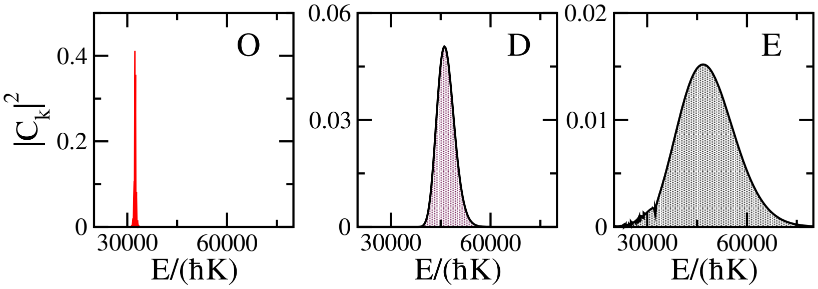

Supplementary Figure 4. Energy distribution of initial coherent states. The coherent states are centered at point O, D, and E, as indicated in the panels; $\xi = 180$, $\hbar = 1$.

The derivation of Γ^2 for the other coherent states is analogous. As evident from the slowest decay of the survival probability for the initial coherent state $|\Psi_O(0)\rangle$ in Fig. 2d of the main text, this state has the smallest variance Γ_O^2. This happens because the corresponding classical point O is a stationary point. The gradient and Laplacian of the Hamitonian vanish at O, so the initial diffusion constant for the Glauber coherent state $|\Psi_O(0)\rangle$ is the smallest one.

In the Supplementary Figure 4, we show the energy distributions of the coherent states $|\Psi_O(0)\rangle$, $|\Psi_D(0)\rangle$, and $|\Psi_E(0)\rangle$. The width of the distribution for $|\Psi_O(0)\rangle$ is significantly narrower than for the other two states, as anticipated in the paragraph above.

Another feature observed in the Supplementary Figure 4 is the difference in the widths of the energy distributions for coherent states $|\Psi_D(0)\rangle$, and $|\Psi_E(0)\rangle$. Even though both initial states have the same energy, coherent state $|\Psi_E(0)\rangle$ is more spread out than $|\Psi_D(0)\rangle$, which explains why the survival probability $S_p^E(t)$ decays faster than $S_p^D(t)$, as seen in the Fig. 2d of the main text.

Supplementary Note 6.2. FOTOC

The same expansion of the propagator $U(t) = \mathrm{e}^{-i\hat{H}t}$ can be extended to the analysis of the short-time evolution of the FOTOC, where one now needs to compute

$$\left\langle \Psi(0) \middle| \left[1 + i\hat{H}t - \frac{\hat{H}^2 t^2}{2}\right] \hat{W} \left[1 - i\hat{H}t - \frac{\hat{H}^2 t^2}{2}\right] \middle| \Psi(0) \right\rangle$$

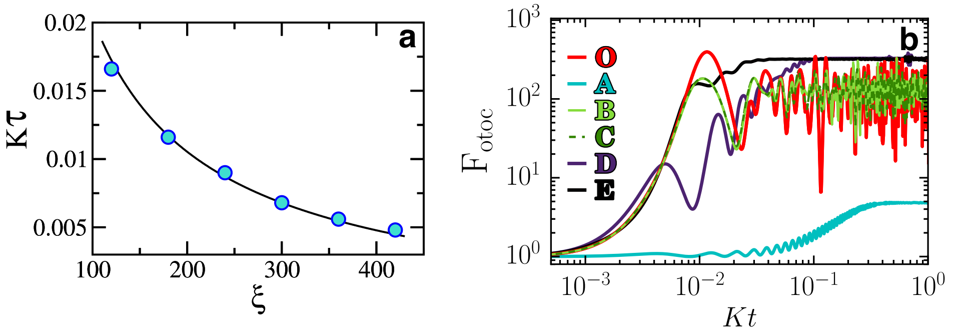

Supplementary Figure 5. Lyapunov time and long-time behavior of the FOTOC. **a** Lyapunov time $\mathcal{T}$ corresponding to the time when the FOTOC for the coherent state $|\Psi_O(0)\rangle$ first reaches its maximum value as a function of ξ. Symbols are for the numerical results and the solid line corresponds to the expression in Eq. (21). **b** Evolution of the FOTOC up to long times for the 6 initial coherent states considered in this work. The highest saturation value is reached by the two initial coherent states with the highest energies, $|\Psi_D(0)\rangle$ and $|\Psi_E(0)\rangle$.

up to $\mathcal{O}(t^2)$ for $\hat{W} = \hat{p}$, $\hat{W} = \hat{p}^2$, $\hat{W} = \hat{q}$, and $\hat{W} = \hat{q}^2$.

For the coherent state O, we find that

$$F_{\text{otoc}}^{(O)} \approx 1 + 8\xi^2 K^2 t^2, \tag{18}$$

so its quadratic behavior holds for

$$Kt < \frac{1}{\sqrt{8}\xi}. \tag{19}$$

Supplementary Note 6.3. Short-time behavior of $\mathbf{M_2(t)}$

To determine the duration of the quadratic behavior of $M_2(t)$, one needs to do the Taylor expansion for each component $C_n(t)$ in Eq. (15), which becomes a tedious exercise even for the coherent state O. This timescale should again be dependent on the value of the control parameter, and we verify numerically that

$$Kt < \frac{1}{\xi}. \tag{20}$$

is an upper bound.

Supplementary Note 7. EXPONENTIAL GROWTH AND INFINITE-TIME AVERAGE OF THE FOTOC

The exponential growth of the FOTOC for the coherent state $|\Psi_O(0)\rangle$ holds up to the Ehrenfest time $\mathcal{T}$ [18], which in our case is given by

$$K\mathcal{T} \sim -0.0027 + \ln(\xi)/(2\xi). \tag{21}$$

In the Supplementary Figure 5a, we show numerical results for $K\mathcal{T}$ as a function of ξ, and we find very good agreement with the expression in Eq. (21). Numerically, the Ehrenfest time is estimated as the point where $F_{\text{otoc}}^{(O)}(t)$ first reaches its highest value.

In the Supplementary Figure 5b, we show results for the FOTOC for the same states shown in Fig. 2b of the main text, but up to longer times. We observe that $F_{\text{otoc}}^{(A)}(t)$ saturates at the smallest value, because $|\Psi_A(0)\rangle$ has the lowest energy. $F_{\text{otoc}}^{(O)}(t)$, $F_{\text{otoc}}^{(B)}(t)$, and $F_{\text{otoc}}^{(C)}(t)$ saturate at an intermediate and very similar value, since the states $|\Psi_O(0)\rangle$, $|\Psi_B(0)\rangle$, and $|\Psi_C(0)\rangle$ have similar intermediate energies. Among these three states, $F_{\text{otoc}}^{(O)}(t)$ fluctuates the most. The infinite-time averages for $F_{\text{otoc}}^{(D)}(t)$ and $F_{\text{otoc}}^{(E)}(t)$ are equal and the highest among the six states, because $|\Psi_D(0)\rangle$ and $|\Psi_E(0)\rangle$ have an equal energy that is higher than that of the other six states. The temporal fluctuations of $F_{\text{otoc}}^{(D)}(t)$ and $F_{\text{otoc}}^{(E)}(t)$ are also much smaller than those for $F_{\text{otoc}}^{(O),(B),(C)}(t)$.

The results in the Supplementary Figure 5b indicate that, despite exhibiting the longest exponential growth and the highest degree of scrambling up to the Ehrenfest time, the initial coherent state $|\Psi_O(0)\rangle$ centered at the hyperbolic point does not maintain the largest degree of spreading at long times. After the Ehrenfest time, $F^{(O)}_{\mathrm{otoc}}(t)$ is surpassed not only by the FOTOC of the state $|\Psi_E(0)\rangle$, which has an overlap with the separatrix, but even by the FOTOC of $|\Psi_D(0)\rangle$, which is away from the separatrix, but has higher energy than $|\Psi_O(0)\rangle$. This raises the question of how to define the notion of "scrambling" and how it depends on the timescales.

SUPPLEMENTARY REFERENCES

[1] M. H. Devoret, Quantum fluctuations in electrical circuits, Les Houches, Session LXIII **7**, 133 (1995).

[2] A. Blais, A. L. Grimsmo, S. M. Girvin, and A. Wallraff, Circuit quantum electrodynamics, Rev. Mod. Phys. **93**, 025005 (2021).

[3] S. M. Girvin, Circuit QED: superconducting qubits coupled to microwave photons, in *Quantum Machines: Measurement and Control of Engineered Quantum Systems: Lecture Notes of the Les Houches Summer School: Volume 96, July 2011* (Oxford University Press, 2014).

[4] N. E. Frattini et al., The squeezed Kerr oscillator: spectral kissing and phase-flip robustness. Preprint at https://arxiv.org/abs/arXiv:2209.03934 (2022).

[5] J. Venkatraman, X. Xiao, R. G. Cortiñas, A. Eickbusch, and M. H. Devoret, Static effective Hamiltonian of a rapidly driven nonlinear system, Phys. Rev. Lett. **129**, 100601 (2022).

[6] N. E. Frattini, U. Vool, S. Shankar, A. Narla, K. M. Sliwa, and M. H. Devoret, 3-wave mixing Josephson dipole element, Appl. Phys. Lett. **110**, 222603 (2017).

[7] B. Wielinga and G. J. Milburn, Quantum tunneling in a Kerr medium with parametric pumping, Phys. Rev. A **48**, 2494 (1993).

[8] J. Venkatraman, R. G. Cortinas, N. E. Frattini, X. Xiao, and M. H. Devoret, Quantum interference of tunneling paths under a double-well barrier (2022), arXiv:2211.04605.

[9] Miguel A. Prado-Reynoso, D. J. Nader, Jorge Chávez-Carlos, B. E. Ordaz-Mendoza, Rodrigo G. Cortiñas, Victor S. Batista, S. Lerma-Hernández, Francisco Pérez-Bernal, and Lea F. Santos, Quantum tunneling and level crossings in the squeeze-driven Kerr oscillator (2023), arXiv:2305.10483

[10] V. Sivak, N. Frattini, V. Joshi, A. Lingenfelter, S. Shankar, and M. Devoret, Kerr-free three-wave mixing in superconducting quantum circuits, Phys. Rev. Applied **11**, 054060 (2019).

[11] T. L. Curtright, D. B. Fairlie, and C. K. Zachos, *A concise treatise on quantum mechanics in phase space* (World Scientific Publishing Company, 2013).

[12] W. H. Zurek and J. P. Paz, Decoherence, chaos, and the second law, Phys. Rev. Lett. **72**, 2508 (1994).

[13] S. Habib, K. Shizume, and W. H. Zurek, Decoherence, chaos, and the correspondence principle, Phys. Rev. Lett. **80**, 4361 (1998).

[14] W. H. Zurek and J. P. Paz, Zurek and Paz reply:, Phys. Rev. Lett. **75**, 351 (1995).

[15] W. H. Zurek, Decoherence and the transition from quantum to classical – revisited (2003), arXiv:quant-ph/0306072.

[16] M. C. Gutzwiller, *Chaos in Classical and Quantum Mechanics* (Springer, New York, 1990).

[17] M. Távora, E. J. Torres-Herrera, and Lea F. Santos, Power-law decay exponents: A dynamical criterion for predicting thermalization, Phys. Rev. A **95**, 013604 (2017).

[18] D. Shepelyansky, Ehrenfest time and chaos, Scholarpedia **15**, 55031 (2020).
